# Beyond the Sociocultural Rhetoric: Female Genital Mutilation, Cultural Values and the Symbolic Capital (Honor) of Women and Their Family in Conakry, Guinea—A Focused Ethnography Among “Positive Deviants”

**DOI:** 10.1007/s12119-022-09975-5

**Published:** 2022-05-11

**Authors:** Marie-Hélène Doucet, Alexandre Delamou, Hawa Manet, Danielle Groleau

**Affiliations:** 1 Division of Social and Transcultural Psychiatry, McGill University, Montreal, QC Canada; 2 Centre National de Formation et de Recherche en Santé Rurale de Mafèrinyah, Université Gamal Abdel Nasser de Conakry, Conakry, Republic of Guinea; 3 Centre National de Formation et de Recherche en Santé Rurale de Mafèrinyah, Conakry, Republic of Guinea; 4 Division of Social and Transcultural Psychiatry, McGill University, Jewish General Hospital, Lady Davis Institute, Montreal, QC Canada

**Keywords:** Female genital mutilation (FGM), Sexuality, Symbolic capital (honor), Cultural values, Positive deviance, Qualitative research

## Abstract

Female genital mutilation (FGM) is justified by sociocultural arguments, including that it guarantees girls’/women’s appropriate sexual behavior, thus preserving family honor. We explored the perspectives of Guineans who do not practice FGM (“positive deviants”), as well as of Guineans who still practice FGM but who are supportive of abandoning the practice (“reluctant adherents”). We conducted a “focused ethnographic” study in Conakry, Guinea with a sample of 58 people. Individual semi-structured interviews were undertaken to explore the views and experiences of 18 women and 12 men of different generations who abandoned the practice of FGM. Group interviews with an additional 16 women and 12 men (half of whom were “positive deviants” and the other half “reluctant adherents”) validated and enriched the data. Participants consider that FGM has deleterious consequences as it: (1) does not prevent girls or married women from being sexually active outside of marriage; (2) may impair couples’ sexual satisfaction, and thus lead to divorce, men’s infidelity or polygamy; and (3) may reduce women's ability to have multiple children, because of the increased risk of infertility or obstetric complications. In addition, participants reported that many Guineans fear that the promotion of FGM abandonment is a Western plot to eradicate their culture. We conclude that Guineans who practice and do not practice FGM share the same cultural values about the importance of culturally appropriate sexual behavior, being married, and having many children, which are central sources of honor (symbolic capital) to women and their families. They, however, have opposing views on how to achieve these objectives. Based on our participants’ perspectives, the harmful consequences of FGM can potentially sabotage these sources of honor. Recommendations for messages aimed at promoting FGM abandonment are discussed.

## Introduction

Female genital mutilation (FGM), commonly known as "cutting" or "excision", refers to various modifications of girls’/women’s genitalia (e.g., removal of the clitoris; stitching of the labia majora; scraping) (WHO, 2020). This two-thousand-year-old tradition (Mackie, 2000) is justified by sociocultural arguments that may vary according to local contexts. It is thought that the most ancient reason for practicing FGM was to enable polygamy, using FGM to reduce women's sexual desire to ensure that (1) wives were faithful to their husbands, and (2) husbands were the biological fathers of their wives' children (Mackie, 2000). FGM has no known medical benefits (WHO, 2020), but carries immediate health threats (e.g., severe pain, hemorrhage) and adverse long-term risks that can impact women physically (e.g., genito-pelvic infections), psychosexually (e.g., painful intercourse), reproductively (e.g., obstetric complications), and mentally (e.g., anxiety) (Andro & Lesclingand, 2016; Berg et al., 2014).

Despite the multiple strategies aimed at promoting the abandonment of the practice, the FGM prevalence remains high in many countries (UNICEF, 2013, 2016). This could be because the implementation of these strategies is situated in a socio-historical context in which many colonialists, religious missionaries, feminists and activists have, based on their own ideological reasoning, attempted to “eradicate” the practice in a way that was not in adequation with the local culture (Gruenbaum, 2005), thus creating resistance. Moreover, some anti-FGM discourses have been criticized for putting forward arguments that were meaningless to people practicing FGM, thus not prompting them to question perpetuating the custom (Barry, 2015). It is therefore essential to find new ways of approaching the issue of FGM abandonment. Although numerous studies have identified the justifications for which communities *practice FGM* (UNICEF, 2013), the focus has not been on the *non-practice of FGM* in high-prevalence countries. Uncovering the sociocultural counter arguments to practicing FGM may inform strategies to ensure that the promotion of FGM abandonment is culturally grounded (Barry, 2015).

### Study Population

In Guinea, FGM is practiced almost universally, with a national prevalence of 95–97% (INS & ICF, 2019; UNICEF, 2016), despite an anti-FGM law and over 30 years of efforts to reduce the practice (Barry, 2017; Gouvernement de la République de Guinée, 2008). The main reasons given by Guineans to justify the practice are respect for the custom handed down by their ancestors, and controlling women's sexuality—before and during marriage—to favor the marriageability of girls and preserve family honor (Barry, 2015, 2019). Strong social pressure to have girls cut prevails in Guinea (Barry, 2015). However, this very high FGM prevalence may not be an accurate reflection of the strength of this apparent norm (Cislaghi & Heise, 2018), as more than a third (37%) of Guinean women (17% of women living in Conakry) are of the opinion that the FGM practice should end (Barry, 2019). Moreover, the perspectives of Guineans who do not practice FGM are unknown. This study aimed to explore the points of view, beliefs and experiences of Guineans who decided to abandon the FGM practice in a context where the FGM tradition is widespread. Based on this understanding, a further aim was to propose culturally grounded recommendations for public health strategies to promote the abandonment of FGM in an effective and sustainable way.

## Methods

### Methodology and Conceptual Framework

We chose the "focused ethnography" methodology (Knoblauch, 2005) to conduct an in-depth exploration of the non-practice of FGM. As this paper presents results complementary to those of an already published paper, refer to Doucet et al. (Doucet et al., 2020) for further details on methodology.

### Theoretical Background

Despite public health efforts and anti-FGM laws, FGM is still highly practiced in many countries (UNICEF, 2013, 2016). It is therefore imperative to seek new evidence-based ways to promote the abandonment of FGM. Focusing on the “non-practice” of FGM in a context of high FGM prevalence is an innovative approach inspired by the notion of “**positive deviance**”, which refers to individuals who do not conform to the prevailing sociocultural norm—i.e., who are “deviants”—and who, as a result, benefit from health advantages—hence the use of the “positive” adjective (Marsh et al., 2004). In other words, they have adopted certain practices and behaviors that are contrary to the cultural norm but that lead to better health outcomes (Nutrition Working Group & CORE, 2003). For our study, the term “deviance” refers to the fact that some people are not complying with the widely-held social expectation of behavior to have their daughters cut. And the term “positive”—taken from an outsider perspective (from the point of view of those who do not practice FGM and/or from the public health perspective)—refers to FGM bearing health risks, its abandonment being therefore beneficial. As a side note, from the insider perspective of people who perpetuate the FGM tradition, those who do not comply with FGM are violating interdependent expectations and, if known to others, is commonly resulting in negative social sanctions (Barry, 2015): in this case, the “deviance” of not complying with FGM is not “positive”. This approach is based on the assumption that solutions to challenges already exist locally (Nutrition Working Group & CORE, 2003), and that public health strategies using arguments from local communities are more likely to be culturally acceptable, and therefore feasible and sustainable (Population Council, 2008). To our knowledge, only one initiative used the “positive deviance” approach to tackle FGM (McCloud et al., 2003) and showed a reduction in the number of girls undergoing the FGM practice (Population Council, 2008). While public health strategies involving "positive deviants" often involve having them serve as role models, the notion of "positive deviance" does not necessarily imply that "positive deviants" have higher social status and visibility. Moreover, since the non-practice of FGM is a rare phenomenon in Guinea, our research approach aimed to include people who were able to enact their decision to not practice FGM despite the prevailing sociocultural context—whether they were leaders in their community or rather discrete (Doucet et al., 2020). We thus used the approach of "positive deviance" to explore the sociocultural arguments underlying the non-practice of FGM.

In addition, knowing that FGM is believed to generate honor and respectability to women as well as to their family as a whole (Barry, 2015), we analyzed respondents’ narrative with the aim of unveiling their views about other sources of honor and respectability that are central for Guinean women and their family, and how these may be impacted by the practice and non-practice of FGM. We thus aimed to build from the concept of **symbolic capital**, by identifying context-dependent sources of honor, dignity and respectability to women and their family (Bourdieu, 1987). For example, in several African societies, married women with children have a higher symbolic capital than those without children (Dyer, 2007). As far as we know, this is the first study using this innovative approach of combining positive deviance and symbolic capital to explore and analyze FGM-related issues.

### Study Setting and Participants

The study took place in Conakry, the capital of Guinea. This urban environment facilitated the recruitment of participants. Thirty "positive deviants" were first interviewed individually. Group interviews were subsequently conducted with 14 “positive deviants”, to validate the data collected through individual interviews, thus increasing the validity of the results by triangulation (Green & Thorogood, 2014). Additional group interviews were undertaken with 14 "reluctant adherents"—i.e., people who are supportive of abandoning the practice but are reluctant to do so due to factors such as social pressure (see the concept of “readiness to change” in Shell-Duncan & Herniund, 2006)—to explore their views about "positive deviants’" arguments. In total, we included 58 participants (Table 1).

**Table 1:** Participants included in the study

	Individual interviews	Group interviews		Total
	"Positive deviants"	"Positive deviants"	"Reluctant adherents"	
Women	18	8	8	34
Men	12	6	6	24
Total	30	14	14	**58**

To be included, “positive deviants” had to firmly disapprove of FGM practice; moreover, parents and grandparents should have at least one uncut daughter/granddaughter. Female participants could be included regardless of if they were cut. We sought to maximize the heterogeneity of the socio-demographic profiles of the individually interviewed participants (Miles et al., 2014) by considering the following criteria: gender; generation—i.e., young adults, parents, and grandparents; level of education; religion; and ethnic identity. For the group interviews, no specific criteria were applied. Women and men were interviewed in separate groups.

Participants were recruited using the “snowball sampling” technique (Miles et al., 2014), with the help of local associations promoting FGM abandonment, people from the community, and study participants. Three persons who were approached refused to participate, stating they feared, among other things, that people around them would criticize them for “following white people”. Minors (under the age of 18) were excluded from the study.

### Data Collection

Individual interviews are an appropriate way to explore complex and sensitive topics such as FGM (Green & Thorogood, 2014). To explore and gain an in-depth understanding of the views, beliefs and experiences of “positive deviants”, we adapted the McGill Illness Narrative Interview schedule (Groleau et al., 2006), which was conceptualized to explore respondents’ health-related subjective meaning and experiences in their sociocultural context. The interviews began with an unstructured account of participants’ personal story about the events that led them to reject the practice of FGM in the sociocultural context of Conakry. This included, when relevant for some women, their personal experience of FGM. We then asked questions to explore: participants’ perceptions of the experience of family members, friends, or colleagues who do not practice FGM, and how it differs from their own experience (analogical reasoning); their opinions about the popular justifications for the FGM practice, and the reasons why they decided to abandon the FGM practice (rational reasoning); their ideas for FGM abandonment strategies; and their socio-demographic characteristics. Recruitment continued until data saturation was reached. Discussions took place in a space chosen by the participants to express themselves freely. Interviews lasted an average of 47 min.

Group interviews allowed “positive deviants” and “reluctant adherents” to discuss the arguments presented by “positive deviants” in the individual interviews about the implications of FGM; the strategies the latter proposed—in terms of their relevance, acceptability, feasibility, efficiency and sustainability; and whether they had additional or complementary views/suggestions. The length of the group discussions averaged 56 min.

Interviews were conducted in the language chosen by the participants, i.e. French, Soussou, Malinké, and Pular. All interviews were audio-recorded, transcribed, and validated to ensure accuracy; verbatim transcripts that were translated were also validated to ensure the accuracy of the translation in terms of linguistic and socio-cultural meanings (Green & Thorogood, 2014). Data were collected in January–February 2019.

### Data Analysis and Interpretation of Results

Thematic analysis was used to identify salient themes emerging from interviews and make sense of the data related to the research objectives (Green & Thorogood, 2014). This process consisted of six steps: (1) transcript reading; (2) thematic coding of the transcripts, using MAXQDA software; (3) identification of recurring themes; (4) grouping of the themes into overarching thematic categories; (5) summary of each overarching theme; and (6) interpretation of the results using the summaries and applying the conceptual framework.

### Rigor and Trustworthiness

The analytical rigor of this study was first ensured by data saturation, consideration of the whole set of data, and identification of recurring themes as opposed to anecdotes (Seale & Silverman, 1997). In addition, attention was given to “intra-coder reliability” when coding the data (by MHD) (Miles et al., 2014). Results were validated with the literature, certain points of view of the participants were verified for biological plausibility, and the interpretation of the novel results was validated by the Guinean co-researchers (AD, HM) acting as “cultural brokers” (“expert checking”) (Miles et al., 2014; Whittemore et al., 2001). Finally, quotes from participants were included in our scientific article allowing readers to judge the credibility of the interpretations (Green & Thorogood, 2014).

### Reflexivity and Positionality

When recruiting participants, the interviewers (MHD and HM) informed potential participants that the ultimate goal of the study was to inform strategies aimed at promoting FGM abandonment in Guinea. Despite this, we are confident in the authenticity of participants’ responses as many of them were already active in fighting against FGM, and as the others’ accounts were consistent with those of these activists. Our confidence in our results is further supported by the fact that HM, being a Guinean, created a climate of trust with interviewees. During the interviews, participants were asked to clarify their statements in order to ensure full understanding of MHD, who, not being Guinean, did not know all the cultural innuendoes (e.g., taboos surrounding FGM or sexuality) (Green & Thorogood, 2014): this positionality was an asset that allowed for more in-depth data collection. However, the fact that MHD is a White Westerner might explain why some people refused to participate.

### Ethical Considerations

Ethics approval was obtained from the Ethics Committee of McGill University (# A00-B39-18B), and from the National Health Research Ethics Committee of Guinea (# 005/CNERS/18). The interviewers informed the participants of the study's objectives and invited them to read the consent form. All participants signed the form to confirm their agreement to participate in the study and have their interviews audio-recorded. Interviews were assigned a unique code to anonymize them. Data was stored in MHD’s computer—which is protected by a password.

## Results

Participants ranged in age from 18 to 69. Their socio-demographic characteristics are presented in Table 2. Participant’s behavior of publicly disclosing their FGM status or not, their experience with social pressure, and the empowering conditions—mainly social and economic capitals—allowing them to enact their decision not to have their daughters undergo FGM are presented in another paper (Doucet et al., 2020). Among those interviewed individually, although they abandoned FGM practice for their daughters/grand-daughters, 15/18 women reported having themselves undergone FGM and 11/12 men reported being married to a woman or having sisters who had undergone FGM. Many of the women who participated in the group interviews spontaneously reported having been cut. The following sections present the emic perspectives of “positive deviants”, i.e., their points of view, beliefs and experiences about the implications of practicing and not practicing FGM in the sociocultural context of Conakry. It should be noted that the “reluctant adherents” did not express points of view or values that were notably different from those voiced by the “positive deviants”.

**Table 2:** Participants’ socio-demographic characteristics

		Individual interviews		Group interviews	
		n = 30	Percentage (%)	n = 28	Percentage (%)
Generation					
	Young adults^a^	8	27		
	Parents^b^	18	60		
	Grandparents	4	13		
Age					
	18–24	6	20	11	39
	25–29	2	7	4	14
	30–39	10	33	6	21
	40–49	8	27	1	4
	50–59	1	3	2	7
	60–69	3	10	0	0
	*Missing*			*4*	*14*
Level of education					
	University – doctorate	1	3	0	0
	University – master	8	27	1	4
	University – bachelor	5	17	12	43
	*Current studies (secondary school)*	*5*	*17*	*2*	*7*
	Secondary or professional training	6	20	6	21
	Primary or no education	5	17	3	11
	*Missing*			*4*	*14*
Religion					
	Muslim	24	80	28	100
	Christian	6	20	0	0
Ethnic identity					
	Malinké	9	30	8	28
	Peul	6	20	10	36
	Soussou	4	13	7	25
	Badiaranké	5	17	0	0
	Kissi	4	13	0	0
	Guerzé	1	3	0	0
	Manon	1	3	0	0
	*Missing*	*0*	*0*	*3*	*11*

^a^ Unmarried women and men aged between 18 and 30 years old

^b^ This category could include mothers-in-law/co-wives or aunts

## Views on the Popular Justifications for the FGM Practice

Participants explained that FGM is practiced in their communities primarily to ensure that girls behave in a sexually appropriate manner—i.e., that they remain virgins until marriage, are faithful to their husbands, and do not prostitute themselves—in the belief that without FGM they would have an insatiable sexual appetite:

*"They put it into the heads of children that […] if your clitoris is not removed at a young age, you may become […] addicted to men, you will always feel the urge to have sex" (young woman).*

*"[People] think that if girls are not cut, they will want to have sex with many man"* (father).

Thus, given that FGM is believed to reduce girls’/women’s libido, it is promoted as a means to preserve the dignity of women and the honor of the family. However, many participants observed that FGM does not prevent "sexual misconduct", which led them to develop a counterargument to these justifications.

### FGM Does not Guarantee Virginity

Participants stated that FGM does not prevent girls from having premarital sex.

*"In our traditions, girls are cut because they want them to arrive virgins to their husbands. In this case, [...] the family is honored. [...] But it turns out that nowadays it is difficult to keep up with this bet. So, why continue this practice, given all the negative consequences we know?*" (father).

*“Others think that [excision] will preserve the girl's virginity. It's the opposite, girls who have been cut lose their virginity quickly after the cutting”* (mother).

### FGM Does not Prevent Infidelity

Several informants witnessed that women who have undergone FGM have several sexual partners, and some even believe that FGM promotes promiscuity in cut women.

*"Eighty percent are excised, 20% are not excised. The majority [are excised]. But infidelity [of women] is in full swing [in Guinea]"* (uncut young woman).

*"The community practices genital mutilation […] to prevent the girl from committing debauchery and […] to preserve the girl's virginity. On the contrary, girls that have been cut quickly lose their virginity soon after being cut […], while a girl who has not been cut, one man is enough for her. […] I think a cut girl will not have sexual pleasure and will be unfaithful because she is looking for sexual pleasure"* (uncut mother).

In other words, some participants believe that many women who have been cut would unconsciously attribute their sexual dissatisfaction to men's incompetence (rather than to the consequences of genital cutting); they would therefore have sexual intercourse with multiple partners in the hope of finding pleasure.

### FGM Does not Prevent Prostitution

Some participants divulged that FGM is not a guarantee against prostitution.

*"I did an investigation [as part of my job]. We realized that in places where prostitution is present, the girls who have been cut are there. [...] This means that people don't have the right reading or the right information about FGM"* (father, activist)*.*

In short, our participants stated that FGM, in addition to being harmful to the health of girls/women, does not bring the expected sociocultural benefits (e.g. abstinence, fidelity):

"*Not only does excision not achieve its goal, but it also harms girls*" (uncut young woman).

### The Solution: Educate Young People About Sexuality

Our participants stated that "excision does not educate girls" to have appropriate sexual behavior according to the standards of their society: *"It is thought that excision prepares girls to be good wives and good mothers for their future homes but it's actually the exact opposite"*. They also explained that young people have virtually no means to adequately learn about sexuality: FGM used to be an integral part of traditional rites of passage to adulthood during which the elders taught women-to-be "how to behave with people", according to sociocultural norms. But as per our participants, these ritual teachings have disappeared in Conakry; the subject of sexuality is not discussed within families; and sexual education is very rarely offered in schools (e.g., in biology classes).

*"Personally, it was only the lessons I was receiving at school and the advice of the teachers that saved me. But not the women who cut me, not my parents, not at all. So I don't see the purpose for which excision was done to me” (woman, group interview).*

They therefore insisted on the importance of providing girls and boys with opportunities for education on sexuality and culturally appropriate behaviors, while eliminating the harmful practice of FGM.

*"A girl who is fortunate enough to have an education […] will have a much better chance of not having a life of debauchery or losing her virginity before marriage, compared to you who have been cut and have not had the privilege of receiving education and guidance. [...] We must educate our children at home, at school, in churches, ... because [otherwise] it will be a lifelong evil” (woman, group interview).*

Participants also stressed the importance of training young people to develop critical thinking skills ("to know the difference between right and wrong") to make the best decisions for and by themselves. Finally, one father suggested that, in addition to education in schools, it might be interesting to use the media (e.g., newspapers, radio, internet) as a means to carry out this kind of education. He also thinks that young people should be educated from the age of 13–14, "before they do their first [sexual] act".

## Experiences with the Long-Term Deleterious Effects of FGM

Participants spoke at length about the sexual, marital and reproductive consequences of FGM they suffer from daily.

### Implications of FGM on Sexuality

The consequences of FGM on sexuality was by far the most discussed concern of our participants. Many women who have been cut reported having painful sexual intercourse, being deprived of sexual pleasure, or feeling no sexual desire.

*"We [women who have undergone FGM] can't stand [too much sex, because] what makes us horny is what they take away from us there [the clitoris]. We are like wood" (mother).*

*"My friend's clitoris has been completely amputated [...]. We were talking about our sexual stories […] she told me a secret. She was crying. She [...] does not feel pleasure. Instead of pleasure, she feels pain. She even told me that if she could imprison her grandmother who cut her [...] she would have done it [...] because her grandmother took a part of her life away from her"* (uncut young woman).

According to the experience of our participants, women's lack of sensation and pleasure caused by FGM also has a negative impact on their partner's satisfaction, because it hinders reciprocity during sexual intercourse:

*"Sleeping with a woman who doesn't feel anything, it's like playing sports. [...] I would rather [not have sex at all] than sleep with these women. You're the only one who has pleasure, the woman, she's not even happy, she doesn't even feel anything. I tell myself that it's not even worth having sex with these women. I tell myself that in sex, there must be pleasure on both sides, there must be a sharing of pleasure between the two partners"* (young man, 30 years old).

*"Men should ask the wise men, the elders, to stop the FGM practice, because it is a practice that does not suit them either. Because if you amputate the sensitive part of the woman, even if you try as hard as you can, you won't find any pleasure.* Interviewer: So FGM also has an impact on men? *Yes, it has an impact on their pleasure. Because pleasure has to be shared, and if it's not really shared.... It has an impact on both"* (mother).

One participant went even further by mentioning that nowadays, uncut girls are valued by men.

*"Uncut girls are treasures we [men] are looking for! Well, I'm married, but today it's a criterion in the selection of women. We prefer uncut girls. It communicates better with them [sexually]”* (father).

### Deleterious Effects of FGM on Marital Relationships

Participants also considered that the impacts of FGM on the sexuality of couples can lead to disappointment, conflict, and sometimes even divorce.

*"If I had a choice, I would have married a woman who was not cut. I admit it. […] Because I really do see a clear difference. […] I had a girlfriend in the past who was not cut [...]"* [implying that he can compare since he had sex with an uncut woman] (father).

*"[Genital mutilation] prevents women from having sexual contact with their husband, even if he needs it, they have no desire. So all this causes divorce or quarrels in the homes"* (mother).

In addition, several female participants attributed FGM as the cause of men’s infidelity and polygamy, and their narratives suggested that they highly dislike these practices.

*"Because here in Guinea, if men prefer to have four wives, two wives, three wives instead of one, it's because of all this. I say, an excised woman and an unexcised woman, their pleasure is not the same. Your husband comes to you, every day you complain, he'll get fed up. He will go and look for another woman who will not complain"* (cut mother).

*"If the husband sees another woman outside the house, she is not cut, he sleeps with her; and you, his wife who is cut, you sleep with that husband; he will end up saying that it is not the same thing. There is a lot of polygamy, and it’s because of this"* (cut mother).

*"When I got married, I did not wonder [if the girl was] cut, [or] uncut. No. And I do not know a single friend who has done that calculation. But our young brothers today who are looking to get married, on the contrary, are keen to have girls who “communicate” [sexually speaking] better with them because it allows at least to… Have you seen, polygamy and all that? It's the consequences of excision […] [A man who marries an uncut woman] will not need to have another wife. No. It will “communicate” better between them. For what reason is he going to look for another woman? For no reason”* (father).

During their interview, several women alluded to the comparison between co-wives in a polygamous household, explaining that women who have undergone FGM are at a distinct disadvantage compared to their uncut co-wives, since the latter are better able to satisfy their husband sexually. This situation can make cut women very possessive and jealous.

*"There's a big difference. The one who is not excised, ... it's for her that the husband will have more feelings. […] People will say that the uncut woman sent her husband to the "marabout". But this woman will say that her power is in her own hands [referring to the sexual pleasure she can give her husband]. We see a lot of this. [...] That's why we say excision is not good: because if you have an uncut rival, she will take your husband away*" (mother).

In addition, many participants felt that young women who have undergone FGM may nowadays have a harder time finding a husband, to the benefit of those with intact genitalia. This also seems to be the case for divorced cut women who wish to rebuild their intimate life:

*"Sometimes even today, when men want to flirt with you, they first want to find out if you have been cut. If you have been cut, they may literally turn their backs on you, they go looking for the one who hasn't been cut. I have seen several cases"* (divorced cut woman).

### Implications of FGM on Reproduction

Participants considered that FGM can affect women's ability to have many children, a highly important issue in the Guinean cultural context. Some participants first explained that FGM could cause infertility, and that women without children are subject to hostile judgements.

*"All those who are cut must be afraid of infection and sterility. [...] Sterility especially. […] In Africa, you must have children. When you get married, you must have a minimum of 3 to 4 children, because if you don't have that in your house... they tell you everything"* (grandmother).

*"It's very sensitive when a woman doesn't have children here [in Guinea]. So when [those promoting FGM abandonment] said that it causes sterility in the woman, it raised awareness among many people. That's a very, very important argument. That's the main argument"* (grandfather).

Several participants also mentioned that childbirth can be very problematic among women who have undergone FGM, further compromising their chances of raising a large family.

*"I had problems during the delivery. One day I told my husband that I didn't want to have any more children because I am tired of giving birth. So my husband took me to his doctor... We didn't live here, we lived in Liberia [where FGM is not practiced]. [...] The doctor said, "So these are the consequences of genital cutting, this is what makes her tired like this. Your wife can't bear many deliveries because it tires her, you have to accept the number of children God will give her and be satisfied with them”. It happened when I was at my third child. Since that day, my husband has forbidden his daughters to be cut"* (mother).

In response to the interviewer who asked whether she observed differences between cut and uncut women in her midwifery practice: *"Yes, yes, a very big difference. A woman who has never been cut, during delivery, her genitals open up, without any wound or anything. And for a woman who has had an excision, especially primiparous women, we have to enlarge [make an episiotomy] so that the child can come”.* Interviewer: "Is it because the scar tissue is too stiff?" *"Yes”.* Interviewer: "Do they have more tears too?" *"More tears, yes”.*

## Fear of the Population that Westerners want to Eradicate African Culture

Given their decision not to have their daughters cut or to advocate against the FGM practice, many participants said that they are being accused of abandoning their African cultures and customs handed down by their ancestors in favor of Western values. Some are even blamed for having "gone White" or are called "uprooted".

*"It hurts when you try to explain to them, when you try to make them understand the seriousness of the practice, and people remain insensitive. And then they tell you: "But this practice has been practiced since the dawn of time. No woman has ever died because of it. And women who have been cut have children without any problem. So what are you telling us? White people invented these ideas. Why should we follow everything people say? It's our culture" "* (father, activist).

*"There are some, when you talk to them, they tell you: "It's related to globalization, Westerners want to come and put in our heads [...] their stories of white people, they want to make us lose our culture" "* (young woman).

*"Our parents, society, think we're getting paid to testify, even though we're not. They refuse to understand what we want to say. When we talk to them or give them a message, they always say, "You have been paid, you have been instrumentalized, you are here to trample on our cultures because you have received money."* Interviewer: "Who do they think put that in your head?" *"White people"* (young woman, activist).

### Keeping Positive African Customs and Eliminating the Bad Ones

Although many of our participants expressed attachment to their ancestral African traditions and values, they were nonetheless very critical of the deleterious effects of the traditional practice of FGM. They therefore stressed the importance of putting an end to this harmful custom, while remaining true to their African identity.

*"We have customs, we have morals that we must continue to follow, because that's us, that's what we are, it's purely African, but FGM must not be part of [our traditions]"* (uncut young woman).

*"Even if it's the tradition bequeathed by our parents, it has to be said that it's bad. We take what is good in tradition and what is good in modernization, and we put it together. And we stop what is not good"* (father, activist).

## Discussion

Our findings show that contrary to the sociocultural rhetoric on FGM, “positive deviants” believe that FGM: does not guarantee virginity nor prevents infidelity or prostitution; has deleterious consequences on the sexuality of women and by rebound effect on that of men, which can potentially lead to divorce or to men’s infidelity and polygamy; can reduce the chances of young women to find a husband; and can affect women's ability to have many children due to the risk of decreased fertility and of increased childbirth complications. Given the value placed on culturally appropriate sexual behavior, marriage and large families in Guinea (including for our participants), what is implicit in our participants’ narrative is that these consequences of FGM can lead to dishonor for women and their family. Thus, even if a priori the dominant popular belief is that FGM increases the respectability of women and of their family, “positive deviants” have opposite beliefs about the effects of FGM. And these effects can potentially lead to a loss of women’s and their family’s main sources of **symbolic capital**. However, a major finding of our study is that the arguments and beliefs presented by "positive deviants" to justify not practicing FGM are just as much rooted in core Guinean **cultural values**, as the pervasive arguments and beliefs commonly put forward to justify and legitimize the FGM practice in the country. Moreover, our participants' narratives underscore that messages promoting FGM abandonment should reflect Guineans’ cultural values and not foreign values. The next sections will discuss implications of these results for guiding the development of a novel culturally anchored approach to promoting FGM abandonment in Conakry.

## FGM and Preserving Women's and Their Family’s Symbolic Capital: Diametrically Opposed Arguments with the Same Cultural Objective

Our data suggest that "positive deviants" share common cultural values with people who practice FGM (Barry, 2015, 2019). Both discourses idealize that girls/women: (1) have sexual behaviors that are consistent with sociocultural expectations—referring mainly to premarital virginity, fidelity of wives, and non-prostitution (Barry, 2015, 2019), (2) are in a (lasting) marital relationship (Barry, 2015), and (3) have several children, all of which constitute sources of respectability for women*.* For family members, social respectability is, among other things, conferred by the virginity status of girls at the time of marriage, which, according to Barry (2015), is a social indication that their father has effective control over their mother, and that the latter can adequately educate their daughters. This honor is conferred to the family on the day of women's marriage (Barry, 2015). These values are related to preserving and enhancing the honor held by women and their families, and thus contribute to their symbolic capital.

However, "positive deviants" and people practicing FGM have opposing views on how to achieve these objectives. Guineans practicing FGM claim that genital cutting allows to reduce girls’ sexual desire—thus ensuring they are virgins until marriage, faithful to their husband, and that they do not practice prostitution—all of which facilitate girls’ marriageability, and fosters social prestige and honor to women and their family (Barry, 2015, 2019; Mackie, 2000). On the other hand, our “positive deviant” participants abundantly put forward the deleterious consequences of FGM on women's sexual behavior, couple relationships, and reproduction, which are central issues that relate to what confers honor upon Guinean women and their family.

### FGM as a Guarantee for Culturally Appropriate Sexual Behavior: A Myth

Our participants argued that women undergoing FGM do not necessarily arrive at marriage as virgins; this change in perception was also recently found in Barry's Guinean study—which included several women favoring FGM perpetuation (Barry, 2019).This study highlighted that in fact, Guinean adolescent girls are sexually active despite that the majority are cut: nearly one-quarter (23%) of them initiate their sexuality before 15 years old, and two-thirds (66%), before their 18th birthday (INS & ICF, 2019). Moreover, Van Rossem and Gage (2009) have objectivized that FGM does not ensure premarital virginity among Guinean women. The belief that FGM would prevent pregnancy before marriage would actually now be one of the main motivations for many Guinean mothers to have their daughters cut (Barry, 2019). In addition, our participants were convinced that FGM does not prevent women from having extra-marital sex or from becoming prostitutes, the latter being also referred to by Barry (Barry, 2015, 2019). In sum, our participants deconstruct the popular premise that FGM would restrict female sexual activity. Some even take the reasoning further by suggesting that FGM causes sexual dissatisfaction in women, leading many to seek multiple partners in the hope of finding sexual pleasure. Based on what is presented above, it seems clear to the social group of “positive deviants” that despite the prevailing belief regarding FGM, women’s and families’ honor cannot be automatically guaranteed by cutting girls, and that FGM could even be counterproductive.

In order to ensure girls/women’s acceptable sexual behavior, our participants highlighted the importance of making sexuality education accessible to the youth, a proposition that was also advocated by Barry (2019). A significant lack of access to adequate and comprehensive information on sexual and reproductive health for young West Africans has in fact been identified as a key issue in need of receiving attention (Crespin et al., 2016). Young Guineans are therefore exploring their sexuality without being informed on how to prevent serious risks, such as unwanted and early pregnancies as well as sexually transmitted infections (Crespin et al., 2016). Such education could help encourage the population to abandon FGM, rather than using FGM as a means to prevent adolescent girls from becoming pregnant.

### The Implications of FGM on Women’s and Men’s Sexuality: the Weakening of Marital Relationships

As seen in other studies, the harmful impacts of FGM on women's sexuality were reported by our participants (Barry, 2019; Esho et al., 2017). Many have also stated that men experience sexual dissatisfaction due to the consequences of FGM in their wives (or sexual partners)—i.e., because of the pain women experience during intercourse, or their lack of desire. However, the impacts of FGM on men's sexuality have been little studied to date (Varol et al., 2015). Although these implications may go unnoticed by many couples—as they may not know how their sexual life would be without FGM or may be unaware of these effects (Mackie, 2000)—our participants reported that these consequences on sexuality have the cascading effect of undermining the harmony of many couples, and may even lead to divorce. Divorce was also found in Egypt to be a consequence of women’s frigidity due to FGM (Population Council, 2008).

Contrary to the assumption that FGM is practiced for polygamy reasons (Mackie, 2000), our participants suggested that nowadays, it is FGM—and the sexual dissatisfaction it entails—that causes men to marry more than one woman. Furthermore, polygamy appears to be disliked by the women interviewed, especially when some co-wives are not cut, because they are perceived as rivals who may be preferred by their husband.

As stated above, the "marriageability" of girls is also often part of the arguments justifying FGM (Barry, 2015, 2019; Mackie, 2000); however, our participants viewed that "non-cutting" does not affect girls’ chances of marriage. This belief that the FGM status is not related to girls’ "marriageability" was also found in other studies conducted in Guinea (Barry, 2019; Van Rossem & Gage, 2009) and Senegambia (Shell-Duncan et al., 2011). Conversely, our participants stated that the girls who have been cut nowadays have a reduced possibility of finding a husband. Men are in fact more likely to have had sexual relations with both cut and uncut women, and may prefer to marry the latter (Almroth et al., 2001).

### FGM and the Reduction of the Ability to Have Many Children

Our participants expressed significant concern about the risk FGM poses to women's fertility. Fertility is indeed highly valued in Africa because, among other things: motherhood confers social status to women; children are an important source of material and financial security; and fertility is inherent in the perpetuation of the lineage (Bonnet & Duchesne, 2016; Dyer, 2007). Having several children is a social norm deeply rooted in the mores of traditional societies (Shaaban & Harbison, 2005) such as in Guinea, where couples may face strong family pressure to procreate (Bonnet & Duchesne, 2016). African women who are infertile or have few children are often ostracized, stigmatized, and faced with loss of social status; they are also at risk of divorce: all this causing them great suffering (Bonnet & Duchesne, 2016; Dyer, 2007; Sciarra, 1994). Infertility is therefore not only a medical problem, but also has deleterious and substantial personal, family and social repercussions, as it threatens the structure of traditional societies (Shaaban & Harbison, 2005). Although the scientific evidence available to date only allows an association between the most invasive cases of FGM and infertility, it is biologically credible that any alteration of genital structures (even if performed by a physician) may lead to physiological changes that may affect reproduction (Almroth et al., 2005). Indeed, infections of the reproductive organs—especially if they are recurrent, poorly treated or untreated—are the main risk factors for infertility, as pathogens can travel up to the uterine tubes, causing inflammation, scarring and tubal occlusion, blocking the movement of eggs or sperm, and resulting in the inability to conceive (Sciarra, 1994). And it is known that genital mutilation increases the risk of bacterial infections of the reproductive system and sexually transmitted infections (Almroth et al., 2005; Andro & Lesclingand, 2016). Various factors increase the susceptibility of developing these reproductive tract infections, including the unsanitary conditions in which FGM is often performed, genital tissue trauma, complications related to the wound-healing process, and the young age of girls at the time of the procedure—since the thinness of the epithelium and the low acidity of the vagina before puberty make them more vulnerable to infection (Almroth et al., 2005; Andro & Lesclingand, 2016). More research should address infertility's biological and social issues in relation to FGM, given the importance for Guineans to have multiple children.

Our participants also reported the very common experience of obstetric complications in women who have undergone FGM, which has been documented in other studies (Andro & Lesclingand, 2016; Barry, 2015; Berg et al., 2014). Mutilated women are at greater risk of deep lacerations/tears, prolonged labor, difficult delivery, and postpartum hemorrhage than those with intact genitals (Andro & Lesclingand, 2016; Berg et al., 2014). Various mechanisms related to anatomical-physiological changes in the reproductive organs are responsible for these increased obstetric risks: for example (and as explained by our midwife participant), incision/excision of the fragile genital mucosa can cause thick, inelastic scar tissue, which may no longer have the propensity to stretch sufficiently and allow the child to come out of the vagina normally, thus causing tearing or obstructed labor (Berg et al., 2014; Jones et al., 1999). These situations can result in a cascade of interventions such as episiotomy, use of forceps, or Caesarean section, and in an increased risk of severe pain, obstetric fistula, or maternal or neonatal death (WHO et al., 2006). Guinean women who have been cut may suffer an increased risk of obstetric complications, which is an additional factor affecting their chance of having many children.

### FGM as a Counterproductive Mean to Foster and Safeguard Women’s and Families’ Symbolic Capital

**Fig. 1:**
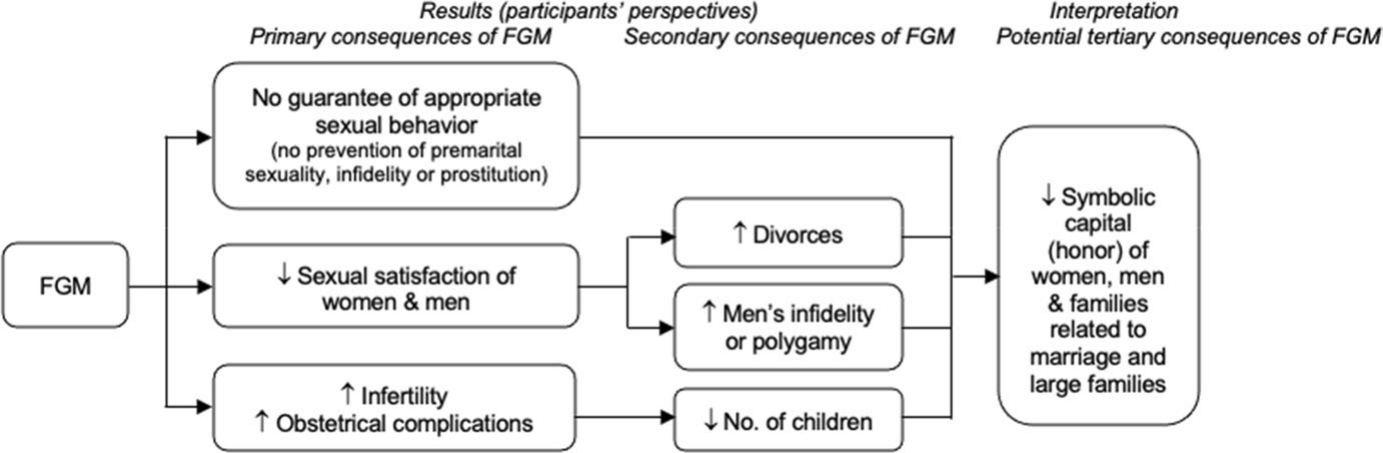
FGM and its sociocultural consequences

In sum, in addition to the immediate consequences of FGM (e.g., intense pain, risk of severe hemorrhage) (Andro & Lesclingand, 2016), practicing FGM imposes multiple burdens to the women who are cut, since they suffer from the long-term risks of mutilation—e.g., painful sexual relations, infertility, difficult childbirth—, and the consequent risks of divorce and of not being able to have as many children as they would want. These secondary consequences of FGM affecting marriage and reproduction not only seem to make women suffer psychologically, we argue that they have deleterious effects on their and their family’s sources of symbolic capital in the honor-based society in which they live (Fig. 1). Indeed, if a priori the predominant belief is that FGM confers symbolic capital to cut girls and their family, the analysis of “positive deviants’” discourse leads to push the reasoning further, to suggest that *in fine*, the consequences of FGM have the potential to sabotage the other central sources of symbolic capital of women/families, such as being married and having large families. FGM would thus miss its sociocultural target of fully insuring the honor of women and families. Therefore, we argue that the non-practice of FGM can promote honor for women and their families through lasting marriages and large families, namely by maximizing the likelihood of experiencing a satisfying sexuality for both women and men, peaceful marriage, optimal fertility and normal deliveries.

## The Fear that Westerners are Trying to Eradicate African Culture by Promoting the Abandonment of FGM

Our data show that many Guineans suspect that "Whites" are funding strategies to combat FGM with the unspoken aim of eradicating their African culture (Johansen et al., 2013). This fear has also been found by activists working with local communities (Behrendt, 2006; HCDH, 2016; Population Council, 2008). The promotion of FGM abandonment in West Africa is situated in a dense historical context, which includes: oppression and country’s disintegration during the colonial era; attempts by missionaries to eradicate local rituals—including the FGM practice—in order to replace them with European practices and ideologies; Western feminists’ discourses that often used offensive and disrespectful terminology; and the continued exploitation of natural resources for foreign interests, while a significant proportion of local people live in poverty (Bouju & De Bruijn, 2008; Gruenbaum, 2005). The Guinean people have thus suffered the burden of hegemonies of all kinds. Therefore, the people’s resistance to any suspicion of "cultural genocide" by neo-colonialism promoting Western values is not surprising (Esho et al., 2011).

Previous strategies implemented to promote FGM abandonment in Guinea have mainly used health, legal and human rights arguments (Barry, 2017), but so far have not significantly reduced the prevalence of the practice (Barry, 2015; INS & ICF, 2019). Indeed, awareness-raising campaigns using health arguments seem to have only shifted the problem, by encouraging the population to turn to healthcare providers to have their daughters cut rather than to traditional excisers (Doucet et al., 2017; HCDH, 2016) given the sociocultural importance of the practice (Shell-Duncan, 2008). Legal methods of repression have most likely contributed to making the practice more clandestine (Doucet et al., 2017; HCDH, 2016). Moreover, the principles underlying the ideology of human rights include equality among all, individualism and autonomy (Howard, 1995; Lindner, 2002); these Western values are a priori contrary to the sociocultural norms and operating modes of collectivist- and honor-based cultures where the community takes precedence over the individual (Lindner, 2002; Triandis, 1989), which predominate in the Guinean society (Doucet et al., 2020). It is, therefore, plausible that Guineans who are not in the process of individualization—most probably the least educated sub-populations (Doucet et al., 2020)—may fear the eradication of their collectivist identity. People exposed to constant global pressures may indeed prefer to seek refuge in the values of their ancestors, which become an affirmation of identity and a protection of their dignity in the face of the intolerable imposition of foreign values (Howard, 1995; Veil, 2007). Consequently, promoting FGM abandonment based on a human rights logic could generate confrontation (Lindner, 2002) and resistance, and even radicalize oppositions (Shell-Duncan, 2008; Veil, 2007), and thus would be counterproductive. Although the protection of girls’/women's right to health, non-torture and life (WHO, 2020) is morally justified, we argue that the Guinean population's sensitivity to the promotion of Western values should be taken into account and that human rights arguments should not be put forward. Rather, like some of our predecessors, we strongly encourage the use of arguments that echo the sociocultural meanings and motivations of Guinean communities (Gruenbaum, 2005), and that highlight their cultural heritage (HCDH, 2016), as they have a greater potential to be better received and to foster reflection leading to abandoning the practice (Barry, 2015).

## Recommendations for Public Health Strategies

Our analysis suggests that FGM abandonment in Guinea should be promoted using Guinean values, instead of reproducing a Western discourse with which Guineans do not identify. As Gruenbaum (2005) wrote, “cultural values can be anchors that reinforce tradition, but they can also be the source of ideas for rethinking and challenging cultural practices. […] [The] same value can be utilized to give meaning to alternative practices […]” (p. 431), such as abandoning FGM. We posit that it would be beneficial to use the logic of fostering the honor of women and their family for public health strategies, as it would offer a better chance of acceptance and impact. Thus, in light of our findings and interpretations, we suggest the following recommendations.

### Rethinking the Content of Messages Aimed at Promoting the Abandonment of FGM

We argue that strategies aimed at promoting FGM abandonment should be designed to use core Guinean cultural values. Namely, (1) put forward that girls’ physical integrity—through the non-practice of FGM—will favor lasting marriages and large families, and thus promote women’s/families’ honor (symbolic capital), and (2) avoid conveying foreign standards—e.g., conveying Western values which may be perceived as a threat to African culture—and focus on positive traditional African values whenever possible. Thus, by being culturally resonant, messages are more likely to be acceptable and sustainable.

The current trend to promote the abandonment of the FGM practice mainly uses health and human rights logics; however, to increase the impact of strategies to promote a change in attitudes and behavior among the Guinean population, we propose a new approach. We advocate that raising awareness of the risks of FGM for women's health—whether the practice is performed by traditional excisers or by physicians—should be maintained, but that messaging should also go beyond the health consequences and focus on the sociocultural values by highlighting the negative implications of FGM (see Fig. 1) and the eventual impact it can have on the symbolic capital of women and families. In particular, this study highlights that nowadays, young men are increasingly seeking to marry uncut girls. Considering that the marriageability of girls is an issue for the status not only of the girl herself but also of the family as a whole, we suggest that this argument be taken into account in campaigns to promote the abandonment of FGM in Guinea. Moreover, messages should emphasize that FGM is likely to affect the quality of couples’ sexual relations and consequently the durability of marriages. However, attention should be paid to ensure that the messages/strategies do not to stigmatize women who have undergone FGM.

These messages should target society as a whole: women and men—as the implications of FGM are not limited to women—and people of all generations—youth, parents/aunts, and grandparents/elders. Finally, the messages should be delivered by Guineans who have credibility in the eyes of the target audiences—such as religious leaders—rather than by outsiders. The effectiveness of this strategy for changing attitudes and behaviors in favor of abandoning FGM should be assessed, and studies should be undertaken to identify who would be the optimal change agents to lead such a strategy.

### Providing Young People with Educational Opportunities on Sexuality and Culturally Valued Behaviors

Our participants suggested there is a pressing need to provide girls and boys with opportunities for education about sexuality and culturally valued behaviors (Barry, 2019). We argue that such education should be evidence-based and comprehensive, enabling young people to acquire the knowledge, attitudes and empowerment (Patton et al., 2016) required to adopt safe sex behaviors, while avoiding the maintenance/reinforcement of gender inequalities. These teachings should be offered from early adolescence (e.g., as young as 10–12 years of age), and adapted to the developmental stages of young people (Patton et al., 2016). These strategies should be socio-culturally acceptable, feasible and affordable, and consider that many young Guineans do not attend school and/or cannot read or write (INS & ICF, 2019). Complementary avenues for education could include: (1) implementing the program in schools (Patton et al., 2016); (2) collaborating with religious leaders*,* given their great power to influence the transformation of sociocultural norms and ancestral values (Crespin et al., 2016); and (3) using media such as television and radio, and new technologies such as the internet ("eHealth") and mobile phones ("mHealth") (Patton et al., 2016). Thus, uncut and "educated" girls would have a better chance of reaching the symbolic capital valued by Guineans, in addition to benefiting from an optimal level of health and well-being.

### Transferability

The results and interpretations of this study were validated for cultural adequacy. Therefore, we believe that our findings and recommendations could be transferable to Guineans who would like to abandon the FGM practice and who live in Conakry. They may also apply to other urban areas of Guinea (Green & Thorogood, 2014; Miles et al., 2014).

### Strengths and Limitations of the Study

Undertaking a “focused ethnographic” study and using the concepts of “positive deviance” and symbolic capital to analyze the data was a very effective and innovative approach to highlighting important sociocultural issues related to the practice/non-practice of FGM in the context of Conakry. In addition, using the logic shift of “positive deviants” as a starting point for informing strategies to promote FGM abandonment is a promising avenue. And although some of the "positive deviants" who were approached declined to participate in our study, we are confident in the validity of our results since we have reached data saturation. However, our study has some limitations. The fact that some people declined to participate in the study due to concerns about sanctions may reflect a possible selection bias, resulting in our sample not reporting all the types of experiences or views related to the non-practice of FGM. Also, although we were able to portray “positive deviants” according to their social, economic and cultural capital (see Doucet et al., 2020), the interview guide did not include the exploration of their personal symbolic capital and the narratives did not allow to portray their profile in this regard. Moreover, since we did not interview the “reluctant adherents” individually, it is not possible to describe their social, economic, cultural and symbolic capital. As these sources of power can greatly shed light on people’s ability to abandon FGM, we recommend that they be explored further in futures studies. In addition, as learning from “positive deviants” might bear limitations, we suggest that future research explore the barriers to change for “reluctant adherents". Finally, given the limited means to undertake this research (since it was a doctoral research project), we could not expand this exploration into rural and forested areas of Guinea. Therefore, we suggest that this research be replicated in all regions of the country to explore whether other sociocultural logics related to the non-practice of FGM are at stake and offer culturally appropriate and convincing counter-arguments to the different local sub-populations (Barry, 2015).

## Conclusions

This focused ethnography has provided an in-depth understanding of the perspectives, beliefs and experiences of women and men of different generations who do not practice FGM in the sociocultural context of Conakry. Our data suggest that the values held by both those who practice and those who do not practice FGM are the same—namely, the desire to foster girls’/women’s and families’ honor/symbolic capital through culturally appropriate sexual behavior, lasting marriages and large families. However, the analysis of “positive deviants’” discourse suggests that the consequences of FGM—including on women’s/couples’ sexual well-being—can potentially sabotage these central sources of symbolic capital of women/families. The findings of this study can inform government policy makers, public health program planners, and community organization leaders to adapt or create culturally grounded strategies for promoting FGM abandonment in Conakry (Guinea) that have the potential to be effective and sustainable.

## Ethical Approval

This study was performed in line with the principles of the Declaration of Helsinki. Approval was granted by the Ethics Review Board of McGill University (# A00-B39-18B) as well as from the National Ethics Committee for Health Research of the Republic of Guinea (# 005/CNERS/18).

## Consent to Participate

Researchers obtained the free consent from participants after explaining and having them read the detailed consent form. Participants were all asked for their assent before recording the interviews.

## Consent to Publish

Consent to publish has been received from all participants.

## Data Availability

The datasets generated and analyzed during the current study are not publicly available, to maintain the anonymity of the persons interviewed. However, sufficient anonymous verbatim extracts are presented in the paper to illustrate the results.
